# Effect of Gabapentin in a Neuropathic Pain Model in Mice Overexpressing Human Wild-Type or Human Mutated Torsin A

**DOI:** 10.3390/life11010041

**Published:** 2021-01-12

**Authors:** Damiana Scuteri, Laura Rombolà, Silvia Natoli, Antonio Pisani, Paola Bonsi, Chizuko Watanabe, Giacinto Bagetta, Paolo Tonin, Maria Tiziana Corasaniti

**Affiliations:** 1 Pharmacotechnology Documentation and Transfer Unit, Preclinical and Translational Pharmacology, Department of Pharmacy, Health and Nutritional Sciences, University of Calabria, 87036 Rende, Italy; damiana.scuteri@unical.it; 2 Preclinical and Translational Pharmacology, Department of Pharmacy, Health and Nutritional Sciences, University of Calabria, 87036 Rende, Italy; laura.rombola@unical.it; 3 Department of Clinical Science and Translational Medicine, University of Rome Tor Vergata, 00133 Rome, Italy; silvia.natoli@uniroma2.it; 4 Department of Systems Medicine, University of Rome Tor Vergata, 00133 Rome, Italy; pisani@uniroma2.it; 5 IRCCS Fondazione Santa Lucia, 00179 Rome, Italy; p.bonsi@hsantalucia.it; 6 Department of Physiology and Anatomy, Tohoku Pharmaceutical University, Sendai 981-8558, Japan; w-chizu@tohoku-mpu.ac.jp; 7 Regional Center for Serious Brain Injuries, S. Anna Institute, 88900 Crotone, Italy; patonin18@gmail.com; 8 School of Hospital Pharmacy, University “Magna Graecia” of Catanzaro and Department of Health Sciences, University “Magna Graecia” of Catanzaro, 88100 Catanzaro, Italy; mtcorasa@unicz.it

**Keywords:** DYT1, torsin A., transgenic mice, neuropathic pain, snl, gabapentin

## Abstract

Background: DYT1 dystonia is the most common form of early-onset inherited dystonia, which is caused by mutation of torsin A (TA) belonging to the “ATPases associated with a variety of cellular activities” (AAA + ATPase). Dystonia is often accompanied by pain, and neuropathic pain can be associated to peripherally induced movement disorder and dystonia. However, no evidence exists on the effect of gabapentin in mice subjected to neuropathic pain model overexpressing human normal or mutated TA. Methods: Mice subjected to L5 spinal nerve ligation (SNL) develop mechanical allodynia and upregulation of the α2δ-1 L-type calcium channel subunit, forming a validated experimental model of neuropathic pain. Under these experimental conditions, TA is expressed in dorsal horn neurons and astrocytes and colocalizes with α2δ-1. Similar to this subunit, TA is overexpressed in dorsal horn 7 days after SNL. This model has been used to investigate (1) basal mechanical sensitivity; (2) neuropathic pain phases; and (3) the effect of gabapentin, an α2δ-1 ligand used against neuropathic pain, in non-transgenic (NT) C57BL/6 mice and in mice overexpressing human wild-type (hWT) or mutant (hMT) TA. Results: In comparison to non-transgenic mice, the threshold for mechanical sensitivity in hWT or hMT does not differ (Kruskal–Wallis test = 1.478; *p* = 0.4777, although, in the latter animals, neuropathic pain recovery phase is delayed. Interestingly, gabapentin (100 mg/Kg) reduces allodynia at its peak (occurring between post-operative day 7 and day 10) but not in the phase of recovery. Conclusions: These data lend support to the investigation on the role of TA in the molecular machinery engaged during neuropathic pain.

## 1. Introduction

Dystonia is a severe hyperkinetic movement disorder involving basal ganglia and cerebellum [1,2]. Similar to other movement disorders, e.g., Parkinson’s and Huntington’s diseases, dystonia also implicates abnormalities of complex sensorimotor integration [3]. In fact, the alteration of the cortico–striatal–thalamic–cortical motor loop is usually preceded by sensory manifestation, such as pain and kinaesthesias; moreover, there is a “sensory trick” (*geste antagonistique*) consisting in a proprioceptive input, not only of psychogenic nature, that is able to improve posture abnormalities [3]. Central processing of sensory input is altered during dystonia [4]. The DYT1 dystonia is a severe early-onset inherited dystonia caused by a GAG deletion (ΔGAG) in the gene TOR1A of torsin A (TA), which is responsible for the loss of a residue of glutamic acid in the carboxy-terminal region [5]. TA is a ubiquitous chaperonine-like “ATPase associated with a variety of cellular activities” (AAA + ATPase) [6] distributed in the striatum, cerebellar cortex, and deep cerebellar nuclei [7]. TA is localized in the endoplasmic reticulum, and it is involved in cytoskeletal dynamics [8], in the endoplasmic reticulum-associated degradation in response to stress [9], and in the release of neurotransmitters [7,10]. The presenting symptom of dystonia is represented by pain in 10% cases of cervical dystonia, the latter being experienced by 66–90% of these patients and by 56–62% of patients suffering from other forms of dystonia [11]. This finds support in the highlighted lowered mechanical threshold to pressure in dystonic patients [12]. Patients with adult-onset focal dystonia affected by pain have been proven to develop more severe neuropsychiatric symptoms in a large international, multicenter study [13]. Pain in dystonia is not exclusively of muscular origin, as demonstrated by the evidence that it is often unrelieved by botulinum toxin, but it can mirror a dysfunction of the descending inhibitory control [14]. For instance, a novel peripherally induced movement disorder, named dancing dorsal quadrilaterals syndrome, is induced by abnormal central (spinal and supraspinal) sensorimotor reorganization due to prolonged neuropathic pain [15]. Apart from pain, dystonia is associated also to other, non-motor, symptoms, in particular psychiatric and behavioral symptoms [16]. These symptoms need accurate assessment, representing important predictors of health-related quality of life, as in neurodegenerative diseases such as Parkinson’s and Alzheimer disease [17]. Lines of transgenic mice overexpressing the human mutant (hMT) or the human wild-type (hWT) TA have been produced as models of DYT1 dystonia [18]. The hMT mice show slower learning and decreased motor activity during exposure to the open field at 9 months of age and also shorter stride length than non-transgenic mice, instead of an overt dystonic behavior [18]; the latter could be explained by the observed difference in the response to amphetamine, without alteration of pre-synaptic transporters or post-synaptic dopamine receptors and, thus, in the release or transport of dopamine [19]. An altered D2 dopaminergic signaling [20,21,22,23,24] that is suggestive of imbalance between dopaminergic and cholinergic neurotransmission in dystonia [25] and linked to disinhibition of striatal GABAergic synaptic activity [26] is displayed by DYT1 dystonia model mice. Incidentally, the D2 receptors dysfunction observed in hMT mice is counteracted by the pharmacological blockade of adenosine A2A receptors [27]. Abnormalities occur in the developmental period most influencing synaptogenesis, as demonstrated in the cerebellum [28]. Indeed, there is premature long-term potentiation (LTP) with increased levels of pro-brain-derived neurotrophic factor (BDNF) and BDNF and the accumulation of α-amino-3-hydroxy-5-methyl-4-isoxazolepropionic (AMPA) receptors in striatal spiny neurons of DYT1 dystonia model mice [29]. Interestingly, also striatal opioid signaling is affected by TA mutation, due to increased levels of mu opioid receptor with stronger inhibition of the firing of cholinergic interneurons [30]. These alterations can be involved in motor function, but it can suggest an influence of TA mutation on pain processing as well. The aim of the present original research is to study the following in mice subjected to neuropathic pain model overexpressing human normal or mutated TA: (1) basal mechanical sensitivity; (2) neuropathic pain phases; and (3) the effect of gabapentin, which is one of the most used drugs against neuropathic pain acting on the α2δ-1 calcium channel subunit, overexpressed during central sensitization and allodynia in pain models [31].

## 2. Results

### 2.1. Basal Mechanical Sensitivity of Mice Overexpressing Human Wild-Type and Mutated TA

**Figure 1:**
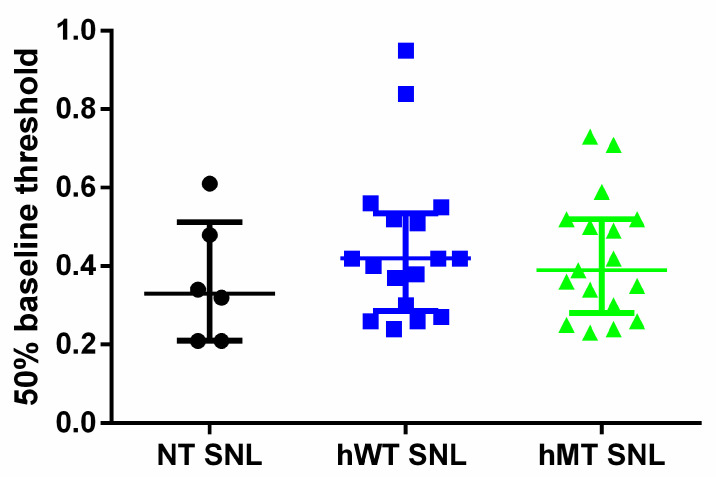
Baseline mechanical sensitivity in non-transgenic (NT) mice and in mice overexpressing human wild-type (hWT) and mutated (hMT) torsin A (TA). hWT and hMT show baseline mechanical sensitivity analogue to NT mice. The overexpression of normal human (hWT) or mutated (hMT) TA does not induce any significant variations of baseline mechanical sensitivity compared to non-transgenic (NT) animals (Kruskal–Wallis test = 1.478; *p* = 0. Data are expressed as median + interquartile range (IQR) of 50% baseline threshold. *p* values < 0.05 are considered statistically significant. n: NT = 6, hWT = 17, hMT = 17.

The purpose of the present original research at first consists in investigating the basal mechanical sensitivity in mice overexpressing human wild-type (hWT) or mutated TA (hMT), as compared to control, C57BL/6 mice (non-transgenic: NT). The baseline level of mechanical sensitivity did not differ in hWT and hMT mice (Figure 1; Kruskal–Wallis test = 1.478; *p* = 0.4777.

### 2.2. Prolonged Mechanical Allodynia in Mice Overexpressing Mutated TA

**Figure 2:**
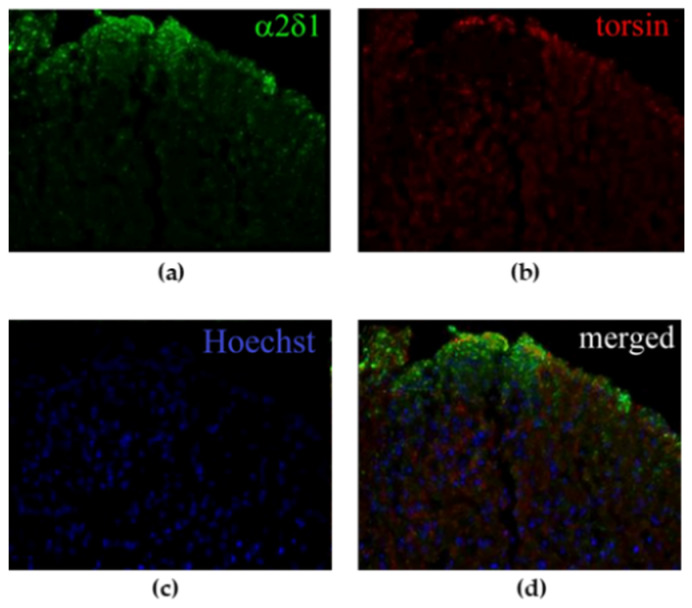
Immunoreactivity in the spinal dorsal horn of spinal nerve ligation (SNL) mice on the 7th day of (**a**) α2δ-1 L-type calcium channel subunit and (**b**) torsin A (TA). (**c**) Nuclei have been counterstained with Hoechst. (**d**) TA and α2δ-1 colocalize in the most superficial *laminae* of the spinal dorsal horn ipsilateral to the ligation (20× magnification).

NT mice subjected to spinal nerve ligation (SNL) show mechanical allodynia of the ipsilateral hindpaw starting from the 3rd post-operative day, reaching its peak between the 7th and 10th day and lasting for 28 days, after which mechanical sensitivity gradually returns to baseline levels. In particular, mechanical allodynia coincides with upregulation of α2δ-1 L-type calcium channel subunit occurring on the 7th post-operative day in the spinal dorsal horn (Figure 2a). Under these experimental conditions, TA colocalizes with α2δ-1 in laminae 1–2 of the spinal dorsal horn, as shown in Figure 2d.

**Figure 3:**
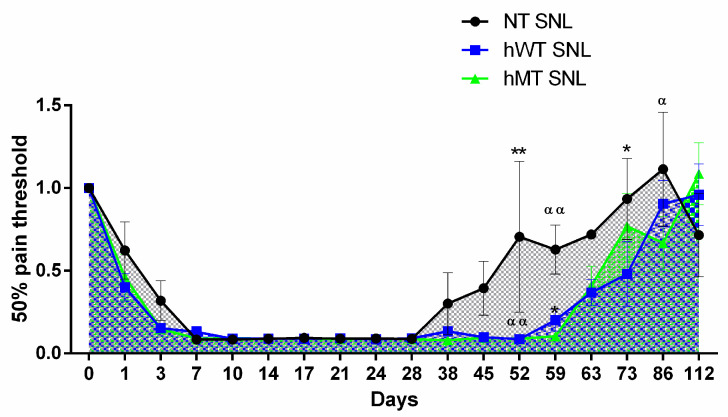
Time-course of spinal nerve ligation (SNL)-induced mechanical allodynia in mice overexpressing human wild-type (hWT) and mutated (hMT) torsin A (TA). Non-transgenic (NT), hWT, and hMT mice do not show statistically significant differences in the development and maintenance of mechanical allodynia. However, hWT and hMT mice present a delayed recovery from sensitization with longer-lasting mechanical allodynia (two-way ANOVA F (32, 374) = 1.561; *p* < 0.05 *; day 52 hWT vs. NT *p* < 0.01 **, hMT vs. NT *p* < 0.01 ^αα^; day 59 hWT vs. NT *p* < 0.05 *, hMT vs. NT *p* < 0.01 ^αα^; day 73 hWT vs. NT *p* < 0.05 *; day 86 hMT vs. NT *p* < 0.05 ^α^). Data are expressed as mean ± SEM of the nociceptive reaction. *p* values < 0.05 were considered statistically significant. n: NT = 4, hWT = 10, hMT = 11.

Both types of transgenic animals do not show differences in the development and maintenance of mechanical allodynia induced by SNL compared to non-transgenic animals, and in the 40 days following surgery, mechanical sensitivity is unchanged in the three different strains, as shown in (Figure 3). On the contrary, hWT and hMT mice present a delay in recovery from sensitization in comparison with NT mice, as shown by the highlighted longer-lasting mechanical allodynia (Figure 3) (two-way ANOVA F (32, 374) = 1.561; *p* < 0.05 *; day 52 hWT vs. NT *p* < 0.01 **, hMT vs. NT *p* < 0.01 ^αα^; day 59 hWT vs. NT *p* < 0.05 *, hMT vs. NT *p* < 0.01 ^αα^; day 73 hWT vs. NT *p* < 0.05 *; day 86 hMT vs. NT *p* < 0.05 ^α^).

### 2.3. Gabapentin Reduces Mechanical Allodynia in the Development but Not in the Recovery Phase in Mice Overexpressing Mutated TA

**Figure 4:**
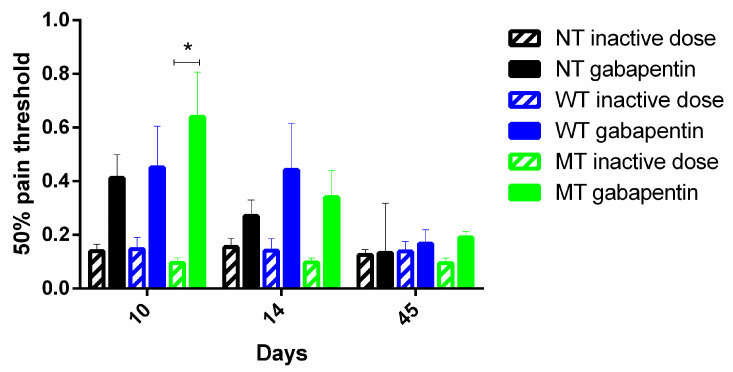
Effect of gabapentin on mechanical pain threshold in. Gabapentin is effective only in the phase of development of mechanical allodynia, but not in the phase of recovery, and its effect is observed in mice overexpressing mutated TA (hMT) (Friedman test = 13.67; *p* = 0.0005; Dunn’s multiple comparisons test: day 10 NT inactive dose vs. hMT gabapentin vs. hMT inactive dose *p* < 0.05 *). Data are expressed as mean ± SEM of the nociceptive reaction. *p* values < 0.05 were considered statistically significant. n: NT inactive dose = 8, NT gabapentin = 9, hWT inactive dose = 3, hWT gabapentin = 6, hMT inactive dose = 3, hMT gabapentin = 10.

NT mice and hWT or hMT mice have been treated with an active (100 mg/Kg ip) or an inactive (1 mg/Kg ip) dose of gabapentin at the peak of mechanical allodynia (10th day after surgery), immediately after (day 14th), and during the recovery phase (45th post-operative day). Gabapentin has been shown to be effective in reducing allodynia at the peak (Friedman test = 13.67; *p* = 0.0005 ***; Dunn’s multiple comparisons test: day 10 NT inactive dose vs. hMT gabapentin vs. hMT inactive dose *p* < 0.05 *), but not after the peak and in the phase of recovery, as shown in (Figure 4).

## 3. Discussion

DYT1 dystonia is a neurodevelopmental disease caused by ΔGAG in the gene encoding TA. In this study, mice overexpressing human wild-type (hWT) and mutated (hMT) TA have been compared with NT mice. The results demonstrate that basal mechanical sensitivity does not significantly differ among the three strains. More importantly, after ligation of the spinal nerve L5 (SNL), transgenic mice present prolonged allodynia, thus suggesting delayed recovery from the sensitization process. According to the original model by Sharma et al., [18] the transgenic hWT and hMT mice show a 1.3–2 fold increase of TA with respect to their non-transgenic counterparts expressing only the endogenous murine protein, as demonstrated through Western blot with D-M2A8 TA-specific monoclonal antibody recognizing both human and murine TA [32]; under these experimental conditions, a more intense TA immunoreactivity with no changes in distribution has been shown [18]. Torsins assemble into dynamic higher-order oligomers to execute their function, but the mutated form of TA could bind to the wild-type, preventing this oligomerization [33]. However, apart from this loss of function, it is possible to hypothesize a gain of function due to oligomerization between human and murine wild-type TA. Most AAA + ATPases present a quaternary structure of rings or spirals, and there is a highly conserved arginine residue called “arginine finger” that is functional to transmission of the conformational changes and to ATP hydrolysis [34]. Therefore, distinct torsins can assembly into higher-order forms in different compositions [34]. In humans, there are four torsin forms among which TA and TB are the most similar, and in mice, the two forms are homologous, and most redundancy occurs at nuclear envelope level [35,36]. More in depth, human and murine TA are highly conserved, presenting 96% similarity [37,38]. Therefore, under our experimental conditions, the theoretical assembly of the cumulative amount of human and murine TA can be involved in the observed results. The aberrant deposition of misfolded proteins, including polyglutamine repeats (i.e., Huntington’s disease) are hallmarks of several neurodegenerative pathologies, e.g., Alzheimer’s and Parkinson’s disease. Torsins have been proven to reduce the phenomenon of polyglutamine repeat-induced protein aggregation, losing this function when mutated [39], thus being potentially protective against neurodegenerative diseases characterized by misfolding proteins. Preliminary evidence suggests that the the upregulation of α2δ-1, seems to colocalize with TA in spinal neurons in the superficial laminae. The latter neurons are known to receive nociceptive signals and are rich in GABA-ergic and glutamatergic interneurons, regulating pain processing to central areas: GABA-ergic inhibition is fundamental after nerve injury to prevent aberrant processing of sensory information within the dorsal horn (see [40]). TA has been found in the glutamatergic and GABA-ergic synapses of the mouse cerebellum [7], and it is involved in endoplasmic reticulum stress, synaptic vesicle cycling, and trafficking, its mutation being associated with abnormalities of neuronal nuclear membrane [41]. In particular, it plays a pivotal role in synaptogenesis [28] and in LTP with increased levels of pro- and mature BDNF and accumulation of AMPA receptors [29]. This might implicate overexpressed and mutated TA on recovery from pain sensitization, a process in which BDNF is fundamentally involved. In fact, its release from activated microglia is responsible for the depolarization shift observed in lamina I neurons during neuropathic pain and allodynia [42]. Moreover, TA mutation influences striatal opioid signaling, being associated with increased levels of mu opioid receptor and stronger inhibition of the firing of cholinergic interneurons [30]. The link between pain and movement disorders is supported by the observation that chronic neuropathic pain can trigger abnormal semirhythmic movements known as dancing dorsal quadrilaterals syndrome [15]. Another important aspect to consider is represented by the possible interaction with α2δ-1. In fact, α2δ subunits alter the release of Ca2 + from the endoplasmic reticulum [40] and likely interact with proteins involved in trafficking [43,44], similar to TA, and this may take part in the effect of gabapentin. Thrombospondins are matrix proteins involved in synaptogenesis [45], bind to α2δ-1, are upregulated after peripheral nerve injury, and disrupt intracellular Ca2 + signaling due to the interaction with this auxiliary subunit of the calcium channel [46]. A better understanding of these phenomena is of the utmost importance to clarify the mechanism of action of α2δ-1 ligands such as gabapentin, pregabalin, and the newest mirogabalin [47,48], which is reportedly endowed with neuropsychiatric activities during chronic pain states [49,50,51], clinical conditions in which, for instance, these drugs are often inappropriately used in non-communicative patients [52,53,54].

## 4. Materials and Methods

Animals: Animal breeding, on a C57BL/6J background (Charles River, catalog number B6JSIFE10SZ-C57BL/6J SPF/VAF; RRID: IMSR_JAX: 000664), and handling were performed in accordance with the guidelines for the use of animals in biomedical research provided by the European Union’s directives and Italian laws (2010/63 EU, D.Lgs. 26/2014; 86/609/CEE, D.Lgs. 116/1992). Based on statistical power analysis and according to similar studies in the literature, the group size has been calculated to balance the need for reliable results while keeping the number of animals as low as possible, in agreement with the 3R approach to refine, reduce, and, at least in part, replace. Different mice strains have been used. In particular, C57BL/6J mice (Charles River, catalog number B6JSIFE10SZ-C57BL/6J SPF/VAF; RRID: IMSR_JAX: 000664), named non-transgenic (NT), have been used as control mice, and mice overexpressing the human wild-type (hWT) and mutated (hMT) TA [18], kindly provided by IRCCS Fondazione Santa Lucia, Laboratory of Neurophysiology and Plasticity, Rome, Italy, have been used to study basal sensitivity and pain processing. For animal reduction, we did attempt to calculate beforehand the sample power by routine formula and setting power to 80% and α = 0.05; however, in view of the lack of pre-existing pain experiments using transgenic animals overexpressing normal or mutated Torsin A, in agreement with the 3R approach, group size has been estimated to balance the need for reliable results while keeping the number of animals as low as possible. According to similar studies in the literature, n = 5 animals per group subjected to gabapentin treatment is sufficient to obtain a 30% reduction of experimentally induced mechanical allodynia with 80% power and α = 0.05. N = 17 animals per each genotype were distinguished in different experimental groups: NT = 6, hWT = 17, hMT = 17 for 50% baseline threshold assessment; NT = 4, hWT = 10, hMT = 11 for experimental pain model; NT inactive dose = 8, NT gabapentin = 9, hWT inactive dose = 3, hWT gabapentin = 6, hMT inactive dose = 3, hMT gabapentin = 10 for pharmacological treatment). The experimental procedures were approved by Fondazione Santa Lucia and University Tor Vergata Animal Care and Use Committees, and the Italian Ministry of Health. Mice have been housed in groups of 4 per cage on a 12 h:12 h light dark cycle at constant room temperature of 22 ± 1 °C and in conditions of relative humidity of the 65% and given food and water ad libitum.

Experimental pain model: the experimental pain model is the spinal nerve ligation (SNL) [55]. Animals have been anesthetized with 2% isoflurane. The surgical procedure consists in a midline incision practiced in the skin of the back at L2–S2 level. The left paraspinal muscles are separated from the spinal and transverse processes at the L4–S1 level. The left L5 spinal nerve is isolated and tightly ligated with 6–0 silk thread and wound sutured. The sham procedure is identical, but the ligation is not performed. The posture of the hindpaw has been subjected to monitoring throughout the post-operative period.

Behavioral Tests: Mechanical allodynia has been assessed through the Von Frey’s test [56]. Behavioral tests have been performed twice a day on the 5th and 3rd day before surgery to assess baseline levels of sensitivity, which are normalized to 1. In the post-operative period, tests have been carried out on the following days after SNL: 1, 3, 7, 10, 14, 17, 21, 24, 28, 37, 45, 52, 59, 63, 73, 86, and 112.

For Von Frey’s test, mice are placed inside Perspex chambers (75 mm × 90 mm) on a wire mesh floor for at least 1 h of acclimation. Calibrated filaments of incremental stiffness (0.41, 0.70, 1.20, 2.00, 3.63, 5.50, 8.50, and 15.10 g), the Von Frey’s hairs (Ugo Basile, Comerio, Italy), are applied to determine the value corresponding to the 50% of the withdrawal threshold. In particular, the 50% response threshold is “50% g threshold = (10 [Xf + kδ])/10.000” where Xf = value (in log units) of the final Von Frey’s hair used; k = tabular value for the pattern of positive/negative responses; δ = mean difference between stimuli expressed in log units. During the behavioral tests, the room temperature and humidity are maintained constant.

Immunofluorescence: anesthetized mice have been transcardially perfused with saline containing heparin followed by 4% paraformaldehyde (PFA) in 0.01 M phosphate buffer (PB, pH 7.4). The thoraco-sacral spinal cord portion has been dissected out, post-fixed in 4% PFA for 2 h, and then transferred into a 30% sucrose solution in PB containing 0.05% sodium azide at 4 °C. The L4–L5 section has been included in O.C.T. (Optimal Cutting Temperature) (Tissue-tek, Sakura) and sectioned at cryostat (Leica CM3050S) into serial slices of 40 μm thickness. The slices have been incubated in free-floating with blocking solution containing PB 0.1 M, 0.3% Triton, 3% normal goat serum, and 2% H2O2 for 1 h at room temperature. After removal of the blocking solution, the slices have been incubated overnight at 4 °C with a primary antibody against TA (polyclonal antibody anti-TorsinA produced in rabbit Abcam ab34540), α2δ-1 (monoclonal antibody anti-α2δ-1 produced in mouse Sigma D219), or the specific neuronal marker NeuN (monoclonal antibody anti-NeuN of rat produced in mouse, Chemicon International MAB377), followed by incubation with the appropriate fluorescent Alexa Fluor 488/568-conjugated secondary antibody (Invitrogen, Carlsbad, CA, USA, A11034/A110364). For α2δ-1, sections have been subjected to antigen retrieval (10 mM citrate buffer, pH 6.0, 0.05% Tween 20, 95 °C for 10 min) [57]. Nuclei have been counterstained with Hoechst (2 μg/mL, Sigma-Aldrich, St. Louis, MO, USA). Controls have been included, omitting the primary antibodies, to assess the presence of non-specific binding of the secondary antibodies. Sections have been mounted and cover slipped with Gel/Mount (Sigma Aldrich, St. Louis, MO, USA). Images have been analyzed by deconvolution microscopy (Leica, EL6000 microsystem; CMSGbH, Mannheim, Germany).

Drug Treatment: Gabapentin is administered intraperitoneally (ip) in two different doses of 1 or 100 mg/kg. Based on the existing literature, the dose of 100 mg/kg has been selected as active dose. Gabapentin is dissolved in depurated water (vehicle) according to its solubility (10 mg/mL). As a result of the reported circadian oscillation of α2δ-1 subunit expression and variability of response to gabapentin, the experiments have started at 9:30 a.m. [58]. The treatment is performed once daily from 7 to 14 days after surgery, i.e., from the peak of allodynia to the beginning of the recovery phase, and the 45th to the 59th day i.e., in the last period of neuropathic pain maintenance. Behavioral tests have been performed 1 h after the administration of gabapentin.

Statistical Analysis: Data, checked for normal distribution (the D’Agostino and Pearson omnibus normality test has been used for samples ≥ 8, whereas for smaller samples, the Friedman test has been used, are expressed as median + interquartile range (IQR) or mean ± SEM and assessed statistically for difference. Kruskal–Wallis and Friedman test followed by Dunn’s multiple comparisons test (in case of null hypothesis rejection) or two-way analysis of variance (ANOVA), followed by Tukey’s multiple comparisons test, were used for non-parametric and parametric data, respectively (GraphPad Prism 6). *p* values < 0.05 are considered statistically significant.

## Data Availability

The whole dataset is included in the manuscript.
